# Reviewer acknowledgement 2012

**DOI:** 10.1186/1471-2377-13-25

**Published:** 2013-04-10

**Authors:** Deesha A Majithia

**Affiliations:** 1 BioMed Central, Floor 6, 236 Gray's Inn Road, London, WC1X 8HB, United Kingdom

## Abstract

**Contributing reviewers:**

The editors of *BMC Neurology* would like to thank all our reviewers who have contributed to the journal in Volume 12 (2012).

## 

Athanasios Petridis

Germany

Inger Marie Skogseid

Norway

Rudiger Seitz

Germany

Rita Horvath

United Kingdom

Omer Karadag

Turkey

Erich Schmutzhard

Austria

Jagarlapudi Mk Murthy

India

Yehonatan Sharabi

Israel

Christian Neri

France

Walter Rachinger

Germany

Jan Kassubek

Germany

Daniele Tomassoni

Italy

Okan Dogu

Turkey

Matthew Dalva

USA

Ombretta Salvucci

USA

Elvira Valeria De Marco

Italy

Merrilee Needham

Australia

Guo-Yuan Yang

China

Michel Modo

USA

Timothy Kwok

Hong Kong

Ettore Beghi

Italy

Richard Camicioli

Canada

Jana Sawynok

Canada

Eduardo Gonzalez-Toledo

USA

Ubaldo Bonuccelli

Italy

Elsdon Storey

Australia

Robert Zivadinov

USA

Moses Elisaf

Greece

Gilles Godet

France

Pritha Ghosh

USA

Christy Tomkins-Lane

Canada

Bianca Weinstock-Guttman

USA

Koichi Hirata

Japan

Jörg Kraus

Austria

Patrick Jung

Germany

Fichet Jerome

France

James Riviello

USA

Gregory Holmes

USA

Burak Yulug

Switzerland

Elizabeth Finger

Canada

Avinoam Safran

Switzerland

Joan Forns

Spain

Peter Albrecht

Germany

Hooman Kamel

USA

Paolo Rossi

Italy

Pablo García-Bermejo

Spain

Matthew Lambon Ralph

United Kingdom

Jonathan Coutinho

Netherlands

Gianfranco Butera

Italy

Inga K. Teismann

Germany

Rajesh Verma

India

J Ivan Lopez

USA

Dimitrios Sokolis

Greece

Agostino Gnasso

Italy

Andrea Lazosky

Canada

Anna Bersano

Italy

Denise A. Valenti

USA

Ziv Gan-Or

Israel

Jean-Michel Rigo

Belgium

Gary Hunter

Canada

Maria Rocca

Italy

David Huang

USA

Victor Tse

USA

Christelle Baunez

France

Angelo Antonini

Italy

Heidi Schambra

USA

Erik Henricson

USA

Vivian Drory

Israel

Graziella Filippini

Italy

Leo Visser

Netherlands

Anna Karin Hedström

Sweden

Paolo Riccio

Italy

Anat Achiron

Israel

Gregor Wenning

Austria

Ulrich Dirnagl

Germany

Veronique H. Flamand

USA

Gad Marshall

USA

Alfonso Lagares

Spain

Liana Rosenthal

USA

Aikaterini Markopoulou

USA

Johannes Dorst

Germany

Jobst Rudolf

Greece

Aurelia Santoro

Italy

Susan Rountree

USA

Adam Handel

United Kingdom

Konstantin Balashov

USA

Christine Belden

USA

Carles Vilarino-Guell

Canada

Ching-Lin Hsieh

Taiwan

Evzen Ruzicka

Czech Republic

Heike Philippi

Germany

Jitendra Sahu

India

Ertugrul Kilic

Turkey

Robert Harvey

United Kingdom

Vittorio Martinelli

Italy

Eyal Muscal

USA

Thomas Scott

USA

Md Akhir Hashmi

India

Bongani Mawethu Mayosi

South Africa

Robert Chen

Canada

Christoph Maier

Germany

Annemerle Beerthuizen

Netherlands

Donald Connor

USA

Chrissa Sioka

Greece

Richard Caselli

USA

Angelo Ghezzi

Italy

Clare Fowler

United Kingdom

Jun Hata

Japan

Nijasri Suwanwela

Thailand

Theresa Green

Canada

Tobias Rupprecht

Germany

Jean Sibilia

France

Giovanni Berlucchi

Italy

Michael Rafii

USA

Francesco Patti

Italy

Károly Orbán-Kis

Romania

Cory Toth

Canada

Bart Van De Warrenburg

Netherlands

Joseph Megyesi

Canada

Mark Mackay

Australia

Linda De Vries

Netherlands

Patrick Kwan

Hong Kong

Balraj Mittal

India

Marco Onofrj

Italy

Joao Gomes

USA

Christopher Ogilvy

USA

Samar Hatem

Belgium

Ada Tang

Canada

Rayaz Malik

United Kingdom

Felix Bokstein

Israel

Guangming Zhu

China

Tobias Struffert

Germany

Joohi Jimenez-Shahed

USA

Seong-Ho Koh

Korea, South

Akira Kunimatsu

Japan

Douglas Jeffery

USA

Tanya Gurevich

Israel

Max Hilz

Germany

Carmel Armon

USA

Shushant Jain

Netherlands

Maja Zivkovic

Serbia

Boel De Paepe

Belgium

Nikolaos Grigoriadis

Greece

Dylan Blacquiere

Canada

Markus Butz

United Kingdom

Alex Zijdenbos

Canada

Alireza Minagar

USA

Louis Caplan

USA

Stefano Seri

United Kingdom

Judith Marcoux

Canada

Blanca Fuentes

Spain

Michael Malek-Ahmadi

USA

Andrew Naidech

USA

Cristina Granziera

Switzerland

Marie Luby

USA

Bogdan Ovidiu Popescu

Romania

Ergun Uc

USA

Ingrid Kane

United Kingdom

**Jerome Engel, Jr**.

USA

Larissa Schwarzkopf

Germany

Euthymia Vargiami

Greece

Monica Saini

Canada

Ted Burns

USA

Beom S. Jeon

Korea, South

Pn Wang

United Kingdom

Domenico Intiso

Italy

Elisabeth Gulowsen Celius

Norway

Annette Förschler

Germany

Francesco Coreaw

Italy

Miguel D'Haeseleer

Belgium

Brenda Banwell

Canada

Bryan Woodruff

USA

Shaheen Hamdy

United Kingdom

Susanna Bacigaluppi

Italy

Beatriz Yadira Salazar Vazquez

USA

Hiroki Ueno

Japan

Sybil Wray

United Kingdom

Corrado Angelini

Italy

Thierry Moulin

France

Yuksel Urun

Turkey

Sandrine De Ribaupierre

Canada

Kentaro Deguchi

Japan

John Ringman

USA

Davud Yapici

Turkey

Frank Huygen

Netherlands

Letizia Leocani

Italy

Sharon Hassin

Israel

Andrew Wong

Australia

Patrick Mcdonald

Canada

Halvor Naess

Norway

Michael Levy

USA

June Halper

USA

Bryan Traynor

USA

Johannes Attems

United Kingdom

Michael Mazya

Sweden

Ruth Luthi-Carter

United Kingdom

Mohammad Rahman Khan

Bangladesh

John Duda

USA

Zohar Eviatar

Israel

Eriks Jankevics

Latvia

Chuan Qiang Pu

China

Keiko Tanaka

Japan

Aleksandra Pavlovic

Serbia

Matthew Huentelman

USA

Randall Woltjer

USA

Raf Brouns

Belgium

George Hines

USA

Christopher Mckevitt

United Kingdom

Richard Doty

USA

Vincent Alvarez

Switzerland

Carolyn Benson

Canada

Niels Hansen

Germany

Li Hu

China

Mohammad Barzegar

Iran

Baruch El-Ad

Israel

William Shankle

USA

Michael Rafii

USA

Szabolcs Szatmari

United Kingdom

Robert Pasnak

USA

Muideen Bakare

Nigeria

Allan Jaffe

USA

Adria Arboix

Spain

Isabelle Arnulf

France

Hans Goebel

Germany

Paola Cicinelli

Italy

Joshua Grill

USA

Jose Cavazos

USA

Jeannette Hofmeijer

Netherlands

Bart Van Der Worp

Netherlands

Richard Chapman

USA

Herta Flor

Germany

Oren Cohen

Israel

Robert Motl

USA

Myla Goldman

USA

Senthil Sundaram

USA

Joseph Quinn

USA

Judith Greer

Australia

Kameshwar Prasad

India

Panitha Jindahra

United Kingdom

Maria Amato

Italy

Roberto Di Bartolomeo

Italy

Erwin Van Wegen

Netherlands

Maren Fedrowitz

Germany

Ingo Helbig

Germany

Myria Petrou

USA

Martijn Muller

USA

Raffi Topakian

Austria

Piero Verro

USA

Narayanaswamy Venketasubramanian

Singapore

Massimo Del Sette

Italy

Robert Hauser

USA

Julian Benito-Leon

Spain

Jan Lewerenz

Germany

Marcus Deschauer

Germany

Michael Dwyer

USA

Joachim Oertel

Germany

Frances Weaver

USA

Fabienne Brilot

Australia

Mario Habek

Croatia

Javier Mar

Spain

Daniel Callan

Japan

Michael Barnett

Australia

Ivan Bodis-Wollner

USA

Michael Linnebank

Switzerland

Rustam Al-Shahi Salman

United Kingdom

Gordon Duncan

United Kingdom

Thomas Spentzas

USA

Dafna Ben Bashat

Israel

Avi Ohry

Israel

Thomas Grau

Germany

Faisal Al Otaibi

Saudi Arabia

Oliver Neuhaus

Germany

Athanasios Covanis

Greece

Arne Lindgren

Sweden

Stefano Palminteri

France

Abhijit Chaudhuri

United Kingdom

Yen-Feng Wang

Taiwan

Emilio Portaccio

Italy

Yara Fragoso

Brazil

John Winer

United Kingdom

Judit Zsuga

Hungary

Peter Hackman

Finland

Nanna Finnerup

Denmark

Istvan Pirko

USA

Hedok Lee

USA

M. Arfan Ikram

Netherlands

Ale Smidts

Netherlands

Satoru Noguchi

Japan

Jacqueline Bernard

USA

Valery Feigin

New Zealand

Armin Rashidi

USA

Ben Seltzer

USA

Jeanne Teitelbaum

Canada

David Rodriguez-Luna

Spain

Menachem Sadeh

Israel

Brian Buck

Canada

Floris Schreuder

Netherlands

Catherine Dotchin

United Kingdom

Rivka Inzelberg

Israel

Masashi Aoki

Japan

Tamara Castillo-Triviño

Spain

Matthew Boyko

Israel

Henriette Klit

Denmark

Andrea Truini

Italy

Karen Fritchie

USA

Claudio Gasperini

Italy

Marcel Post

Netherlands

Roy Beekman

Netherlands

Carol Di Perri

Italy

Huu Phuc Nguyen

Germany

Rashedul Hassan

Bangladesh

Maarten Titulaer

Spain

Svetlana Lorenzano

USA

Aldobrando Broccolini

Italy

Michael Geschwind

USA

Kenneth Tyler

USA

Laszlo Vutskits

Switzerland

Laura Baker

USA

Amiram Catz

Israel

Sachin Patil

USA

Ville Dorothee

France

Giuliana Galassi

Italy

Sarlota Mesaros

Serbia

Jolijn Kragt

Netherlands

Eli Wertman

Israel

Bernardino Fernández Calvo

Spain

Harry Mcnaughton

New Zealand

Yuichi Imanaka

Japan

Paola Torelli

Italy

Cecilie Johannessen Landmark

Norway

Stephen Read

Australia

Peter Kaplan

USA

Eiichi Nomura

Japan

Yuehui Yin

China

Maromi Nei

USA

Shejun Feng

China

Simona Luzzi

Italy

Ehud Mendel

USA

Kenji Hachisuka

Japan

Christine Kremer

Sweden

Gian Marco De Marchis

Switzerland

Berneet Kaur

USA

Samir Amr

Saudi Arabia

Vicente Iragui

USA

Mario Campero

Chile

Wk Chong

United Kingdom

Bodo Laube

Germany

Weerasak Muangpaisan

Thailand

Monika Bekiesinska-Figatowska

Poland

Timothy Kleinig

Australia

Eva Havrdova

Czech Republic

Ralph Benedict

USA

Stefan Oberndorfer

Austria

David Eidelberg

USA

Nathalie Goemans

Belgium

Manoj Sivan

United Kingdom

E. Ann Yeh

USA

Pierre Mégevand

USA

Meir Kestenbaum

Israel

Thomas Leist

USA

Roberto Federico Villa

Italy

Marian Simka

Poland

Inga Zerr

Germany

Ignacio Mata

USA

Robert Fekete

USA

Marcia Pradella-Hallinan

Brazil

Yadira Valles

USA

Felix Fluri

Switzerland

Friedhelm Sandbrink

USA

Peter Berlit

Germany

Giorgio Iervasi

Italy

Carl Cotman

USA

Asa K Wallin

Sweden

Bence B Gunda

Hungary

Laszlo Bajnok

Hungary

Per Wester

Sweden

Mayela Rodríguez-Violante

Mexico

Mario Clerici

Italy

Dimitrios Zafeiriou

Greece

Julien Cassereau

France

Erwin Stolz

Germany

Alan Apter

Israel

Diana Paleacu

Israel

Carmine Vecchione

Italy

Marcus D Souza

Switzerland

Raúl Pelayo

Spain

Natale Daniele Brunetti

Italy

Sarosh Irani

United Kingdom

Nora Fernandez Liguori

Argentina

Toshitaka Umemura

Japan

Veriano Alexandre

Brazil

Marina De Tommaso

Italy

Julie Chandler

USA

Dwight German

USA

Teneille Gofton

Canada

Kai Kisand

Estonia

Jarmo Jääskeläinen

Finland

Antonella Biasiotta

Italy

Edward Zamrini

USA

Kirti Saxena

USA

Brett Stacey

USA

Gabriel Tender

USA

Nicolaas Bohnen

USA

Howard Kirshner

USA

Kjell Flekkoy

Norway

Hugues Chabriat

France

Garth Nicholson

Australia

Jay-Jiguang Zhu

USA

Vladimir Trkulja

Croatia

Arlene Schmid

USA

Christian Neri

France

Giuseppe Lanzino

USA

Paolo Zamboni

Italy

Patrick Sleiman

USA

Jose Cabassa

USA

Jonathan Kleen

USA

Ingeborg Bosma

Netherlands

Carina Wattmo

Sweden

Robert Lindenberg

Germany

Dagmar Nolte

Germany

Robert Silbergleit

USA

Christine Comer

United Kingdom

Alberto Costa

Italy

Rejko Krüger

Germany

Mathew Reeves

USA

Oscar Fernandez

Spain

Nai-Shin Chu

USA

Judith Aharon-Peretz

Israel

Chii-Min Hwu

Taiwan

Jens Schmidt

Germany

Lisa Scalfone

USA

Domenico Italiano

Italy

Jian He

China

Francis Farhadi

USA

Bruce Campbell

Australia

Giovanni Pavesi

Italy

Gabriel Pardo

USA

Eneida Mioshi

Australia

Ferdinand Serracino-Inglott

United Kingdom

Sergei Fedorovich

Belarus

Takahisa Tateishi

Japan

Li Ling

China

## Pre-publication history

The pre-publication history for this paper can be accessed here:

http://www.biomedcentral.com/1471-2377/13/25/prepub

